# Therapeutic Potential of ^47^Sc in Comparison to ^177^Lu and ^90^Y: Preclinical Investigations

**DOI:** 10.3390/pharmaceutics11080424

**Published:** 2019-08-20

**Authors:** Klaudia Siwowska, Patrycja Guzik, Katharina A. Domnanich, Josep M. Monné Rodríguez, Peter Bernhardt, Bernard Ponsard, Roger Hasler, Francesca Borgna, Roger Schibli, Ulli Köster, Nicholas P. van der Meulen, Cristina Müller

**Affiliations:** 1 Center for Radiopharmaceutical Sciences ETH-PSI-USZ, Paul Scherrer Institute, 5232 Villigen-PSI, Switzerland; 2 Laboratory of Radiochemistry, Paul Scherrer Institute, 5232 Villigen-PSI, Switzerland; 3 Department of Chemistry and Biochemistry University of Bern, 3012 Bern, Switzerland; 4 Laboratory for Animal Model Pathology (LAMP), Institute of Veterinary Pathology, Vetsuisse Faculty, University of Zurich, 8057 Zurich, Switzerland; 5 Department of Radiation Physics, The Sahlgrenska Academy, University of Gothenburg, 41345 Gothenburg, Sweden; 6 Department of Medical Physics and Medical Bioengineering, Sahlgrenska University Hospital, 41345 Gothenburg, Sweden; 7 SCK.CEN, Belgian Nuclear Research Centre, BR2 Reactor, 2400 Mol, Belgium; 8 Department of Chemistry and Applied Biosciences, ETH Zurich, 8092 Zurich, Switzerland; 9 Institut Laue Langevin, 38042 Grenoble, France

**Keywords:** ^47^Sc, ^177^Lu, ^90^Y, radionuclide therapy, SPECT, folate receptor, DOTA-folate, IGROV-1 tumor xenografts, preclinical therapy

## Abstract

Targeted radionuclide therapy with ^177^Lu- and ^90^Y-labeled radioconjugates is a clinically-established treatment modality for metastasized cancer. ^47^Sc is a therapeutic radionuclide that decays with a half-life of 3.35 days and emits medium-energy β^−^-particles. In this study, ^47^Sc was investigated, in combination with a DOTA-folate conjugate, and compared to the therapeutic properties of ^177^Lu-folate and ^90^Y-folate, respectively. In vitro, ^47^Sc-folate demonstrated effective reduction of folate receptor-positive ovarian tumor cell viability similar to ^177^Lu-folate, but ^90^Y-folate was more potent at equal activities due to the higher energy of emitted β^−^-particles. Comparable tumor growth inhibition was observed in mice that obtained the same estimated absorbed tumor dose (~21 Gy) when treated with ^47^Sc-folate (12.5 MBq), ^177^Lu-folate (10 MBq), and ^90^Y-folate (5 MBq), respectively. The treatment resulted in increased median survival of 39, 43, and 41 days, respectively, as compared to 26 days in untreated controls. There were no statistically significant differences among the therapeutic effects observed in treated groups. Histological assessment revealed no severe side effects two weeks after application of the radiofolates, even at double the activity used for therapy. Based on the decay properties and our results, ^47^Sc is likely to be comparable to ^177^Lu when employed for targeted radionuclide therapy. It may, therefore, have potential for clinical translation and be of particular interest in tandem with ^44^Sc or ^43^Sc as a diagnostic match, enabling the realization of radiotheragnostics in future.

## 1. Introduction

Presently, the standard treatment of cancer involves surgery, external beam radiation therapy, and chemotherapy. Depending on the tumor type, more specific treatments, including hormonal therapy or immunotherapy, are also viable options [1]. Most attention is currently drawn to the approach of personalized medicine, where the cancer treatment chosen is dependent on patient- and tumor-specific characteristics [2]. Among these is also the concept of tumor targeting to deliver highly toxic agents, specifically to cancer cells. This option requires the design of tumor-specific ligands (tumor targeting agents) which do not—or only at insignificant levels—accumulate in healthy normal tissue. The goal of this concept is to maximize the therapeutic efficacy of the cytotoxic agent and, at the same time, reduce undesired off-target toxicity.

Tumor-targeted radionuclide therapy has emerged as a promising approach for the treatment of disseminated cancer [3]. β^−^-particle-emitting radionuclides are most often used for this purpose. The tissue range of β^−^-particles covers hundreds of cell diameters, hence, β^−^-radiation affects not only cells expressing the target structure, but also neighboring tumor tissue. This phenomenon is known as the “cross-fire effect” and enables the treatment of medium-sized and larger lesions, even if the target is heterogeneously expressed [4]. Currently, ^177^Lu (T_1/2_ = 6.65 days; Eβ^−^_av_ = 134 keV) is the most commonly employed β^−^-particle-emitting radiometal for targeted radionuclide therapy using peptide- or small-molecule-based targeting agents (Table 1) [5].

^90^Y is another radiometal applied clinically, which decays with a half-life of 2.67 days by the emission of β^−^-particles of a relatively high energy (Eβ^−^_av_ = 934 keV), resulting in a maximum tissue range of 10 mm (Table 1). It is, therefore, suitable for the treatment of larger metastases and has been extensively utilized with somatostatin analogs for the treatment of neuroendocrine tumors [6,7]. On the other hand, these properties also contribute to undesired side effects, particularly hematological and kidney toxicities when using ^90^Y-labeled compounds [8,9]. ^177^Lu-based therapy outperformed the use of ^90^Y-labeled compounds in terms of minimizing side effects [10]. The medium-energy β^−^-particles emitted by ^177^Lu affect non-targeted tissues to a lesser extent due to its shorter tissue range (maximum tissue range: ~1.5 mm) [11]. A further advantage of ^177^Lu over ^90^Y is the co-emission of γ-radiation (Table 1), which can be used for the monitoring of tissue distribution and for pre-therapeutic dosimetry using SPECT.

^47^Sc is a novel radionuclide with decay properties comparable to those of ^177^Lu, as it emits medium-energy β^−^-particles (Eβ^−^_av_ = 162 keV) and γ-radiation (Eγ = 159 keV) suitable for SPECT imaging (Table 1) [12,13]. ^47^Sc decays with a half-life of 3.35 days, which may be suitable for small-molecular-weight ligands, considering their rapid pharmacokinetics. The therapeutic potential of ^47^Sc has been shown previously in a pilot preclinical study using ^47^Sc-labeled folate [14]. It can be used towards a theragnostic approach in tandem with ^44^Sc or ^43^Sc as a diagnostic match. ^44^Sc (T_1/2_ = 4.04 h [15]; Eβ^+^_av_ = 632 keV) and ^43^Sc (T_1/2_ = 3.89 h; Eβ^+^_av_ = 476 keV) are radionuclides suitable for PET imaging, due to their favorable physical properties, which makes them interesting for clinical translation [16,17,18]. Preliminary data from clinical applications are available with generator- and cyclotron-produced ^44^Sc, where it was used in combination with prostate specific membrane antigen (PSMA) ligands and somatostatin analogues, respectively [19,20,21].

The goal of this study was to investigate the potential of ^47^Sc for targeted radionuclide therapy and to compare its therapeutic properties with those of ^177^Lu and ^90^Y. For this purpose, we used a DOTA-functionalized folate conjugate for folate receptor (FR)-targeted radionuclide therapy of ovarian cancer, which is known to express the folate receptor with the highest frequency (~90% of cases) [22]. The folate conjugate was labeled with ^47^Sc, ^177^Lu, and ^90^Y, respectively, and the radiometal complexes were investigated extensively in vitro and in vivo. In this study, ovarian IGROV-1 tumor cells were used instead of the commonly-employed cervical KB tumor cells, because they overexpress the FR at levels that reflect the clinical situation more accurately than is the case for KB cells [23]. The therapeutic effects of various activities of ^47^Sc-, ^177^Lu-, and ^90^Y-labeled folate conjugates were investigated in vitro. Biodistribution studies were carried out in IGROV-1 tumor-bearing mice using ^177^Lu-folate, in order to calculate the mean absorbed dose to the tumors and kidneys of mice when treated with ^47^Sc-, ^177^Lu-, and ^90^Y-folate. Subsequently, the therapy experiment was performed using activities which resulted in equipotent tumor doses of ^47^Sc-, ^177^Lu-, and ^90^Y-folate.

## 2. Materials and Methods

### 2.1. Radionuclides

^47^Sc was produced using the ^46^Ca(n,γ)^47^Ca→^47^Sc nuclear reaction, as previously reported [12,14]. ^46^Ca targets were irradiated in the high thermal neutron flux reactor at the Institut Laue Langevin (ILL; Grenoble, France) or at the BR2 reactor at SCK.CEN (Mol, Belgium) and ^47^Sc separated from the target material at the Paul Scherrer Institute (PSI; Villigen-PSI, Switzerland). In the course of these studies, the production and separation processes of ^47^Sc were optimized with the use of ^46^CaO targets instead of ^46^Ca(NO_3_)_2_ targets. ^46^CaO targets (^46^Ca, 5% enrichment, Trace Sciences, Wilmington, DE, USA) were irradiated and the separation method was adapted to enable target dissolution and efficient separation of ^47^Sc from the target material and environmental impurities (Supplementary Materials, Figure S1). After separation, ^47^Sc was obtained as ^47^ScCl_3_ in HCl (0.05–0.1 M).

No-carrier-added ^177^Lu (^177^LuCl_3_/HCl 0.04 M) was kindly provided by Isotope Technologies Garching (ITG GmbH, Garching, Germany) and ^90^Y (^90^YCl_3_/HCl 0.05 M) was obtained from Medeo AG (Schöftland, Switzerland).

### 2.2. Preparation of ^47^Sc-Folate, ^177^Lu-Folate, and ^90^Y-Folate

The DOTA-functionalized folate conjugate (referred to as cm10), was previously developed at the Center for Radiopharmaceutical Sciences at PSI [24]. The radiolabeling of the folate conjugate with ^47^Sc, ^177^Lu, and ^90^Y was carried out at pH 4.5 using HCl (0.05 M, pH 1) and Na-acetate (0.5 M, pH 8), as previously reported for ^177^Lu-folate [24]. The reaction mixture was incubated for 10–15 min at 95 °C, followed by quality control using HPLC (Supplementary Materials). The folate radioconjugates were obtained at high radiochemical purity (>97%) and used without additional purification. Sodium diethylenetriaminepentaacetate (Na-DTPA) was added to the injection solution for complexation of potential traces of unreacted radionuclide.

### 2.3. Tumor Cell Culture and Internalization Experiments

IGROV-1 cells (human ovarian carcinoma cell line) were gifted from Dr. Gerrit Jansen (Department of Rheumatology, Free University Medical Center, Amsterdam, The Netherlands). These cells were cultured in folate-deficient RPMI medium (FFRPMI, Cell Culture Technologies GmbH, Gravesano, Switzerland) supplemented with 10% fetal calf serum (FCS), l-glutamine and antibiotics. Routine cell culture was performed twice a week using trypsin-EDTA (0.25%, Gibco) for detachment of the cells.

Tumor cell uptake and internalization studies were performed using FR-positive IGROV-1 cells as previously reported [25]. The radiofolates were prepared at a molar activity of 10 MBq/nmol and added to IGROV-1 tumor cells in the presence and absence of folic acid, followed by incubation of the cells for 4 h. A detailed description of the procedure is given in the Supplementary Materials. The total uptake and the internalized fraction were calculated as a percentage of total added activity. Each experiment was performed in triplicate.

### 2.4. Cell Viability Assay

^47^Sc-, ^177^Lu- and ^90^Y-folate, prepared at a molar activity of 10 MBq/nmol, were evaluated with regard to their effect on cell viability using a 3-(4,5-dimethylthiazol-2-yl)-2,5-diphenyltetrazolium bromide (MTT) assay as previously reported [25]. IGROV-1 tumor cells were seeded in 96-well plates at 2.5 × 10^3^ cells per well in a volume of 200 μL FFRPMI medium (with supplements). The well plates were incubated at 37 °C and 5% CO_2_ overnight to allow cell adhesion and growth. The following day, supernatants were removed and either only FFRPMI medium (200 μL, without supplements) or medium containing ^47^Sc-, ^177^Lu-, or ^90^Y-folate, at variable concentrations (1.0–40 MBq/mL), was added to each well. The supernatants were removed after 4 h incubation at 37 °C and cells were washed with PBS (200 µL). FFRPMI medium with supplements was added to each well. After an incubation period of 6 days, the cell viability was determined using the MTT reagent and a microplate reader (Victor X3, Perkin Elmer, Waltham, MA, USA) to determine the absorbance of the violet color at 560 nm, as previously reported [26]. To quantify cell viability, the percentage of the absorbance of treated cell samples was compared to the absorbance of untreated control cell samples (= 100% viability). Graphs were prepared using GraphPad Prism software (version 7.0) (GraphPad Software, Inc., San Diego, CA, USA).

### 2.5. Animal Experiments

All applicable international, national, and/or institutional guidelines for the care and use of animals were followed. In particular, all animal experiments were carried out according to the guidelines of Swiss Regulations for Animal Welfare. The preclinical studies have been ethically approved by the Cantonal Committee of Animal Experimentation and permitted by the responsible cantonal authorities (N° 75545, November 2013 and N° 79692, January 2017). Female CD1 nude mice aged 5 weeks were purchased from Charles River Laboratories (Sulzfeld, Germany). All animals were fed with a folate-deficient rodent diet (ssniff Spezialdiäten GmbH, Sulzfeld, Germany), starting one week prior to the injection of the radiofolates.

### 2.6. Biodistribution Studies

Biodistribution studies were performed in triplicate two weeks after IGROV-1 tumor cell inoculation (5 × 10^6^ tumor cells in 100 µL of PBS), as previously reported for other tumor mouse models [24]. ^177^Lu-folate (3 MBq, 1 nmol/mouse) was injected in a volume of 100 μL into a lateral tail vein. The animals were sacrificed at various time points between 1 h and 10 days after administration of the radioconjugate. Selected tissues and organs were collected, weighed, and the activity was determined using a γ-counter (Perkin Elmer, Wallac Wizard 1480). The results were listed as a percentage of the injected radioactivity per gram of tissue mass (% IA/g), using counts of a defined volume of the original injection solution measured at the same time.

### 2.7. Dosimetric Calculations

Dosimetric calculations were performed for all three radiofolates using the experimentally-determined tissue distribution data of ^177^Lu-folate, under the assumption that the radiofolates would distribute equally irrespective of whether ^47^Sc, ^177^Lu, or ^90^Y was coordinated. The data of the ^177^Lu-folate biodistribution were converted to non-decay-corrected values using the half-lives of ^47^Sc, ^177^Lu, and ^90^Y, respectively. The mean specific absorbed dose (Gy/MBq) to the tumor xenografts and kidneys was calculated by multiplication of the time-integrated activity concentration with the emitted electron energy per decay, the absorbed fraction of the emitted electron energy, and a factor of 1.6 × 10^−7^ for converting the unit into Gy/MBq [27]. The absorbed fractions for the tumors (40−50 mg) at the time of injection and the kidneys (~125 mg) were assessed by Monte Carlo simulations using PENELOPE-2014 [28]. Uniform activity distribution within the kidneys and the tumors was assumed for the simulations. The average electron energy emitted per decay for ^177^Lu was 147 keV, for ^47^Sc 162 keV, and for ^90^Y 934 keV, according to the National Nuclear Data Center, Brookhaven National Laboratory USA (https://www.nndc.bnl.gov/mird/, accessed in 2017). The contributions of the emitted photons to the absorbed doses were neglected [29].

### 2.8. Tumor Therapy Studies

The mice were subcutaneously inoculated with 5 × 10^6^ IGROV-1 tumor cells (in 100 µL PBS) into the right shoulder region 4 days prior to the injection of the radiofolates. Each group consisted of 6 animals. Control animals were injected intravenously with saline. The mice received 12.5 MBq (1 nmol) ^47^Sc-folate, 10 MBq (1 nmol) ^177^Lu-folate, and 5 MBq (1 nmol) ^90^Y-folate, respectively. Tumor volumes and body weights of mice were monitored three times a week throughout the study. Endpoint criteria were defined as weight loss of >15% of the initial body weight (at Day 0), tumor volume >1000 mm^3^, ulceration or bleeding of the tumor xenograft or abnormal behavior indicating pain or unease of the animal. The tumor volume (V) was determined with the equation [V = 0.5 × (L × W^2^)], where L is the longest axis and W is the perpendicular axis to L, as previously reported [30]. The results of the tumor growth were presented as the average of absolute tumor volumes of each group. The body weight was presented as a relative body weight (RBW), calculated according to the equation RBW = W*_x_*/W_0_; with W*_x_* = body weight at Day *x* and W_0_ = body weight at Day 0. The relative tumor volume (RTV) was calculated according to the equation RTV = V*_x_*/V_0_; with V*_x_* = tumor volume at Day *x* and V_0_ = tumor volume at Day 0. The therapeutic efficacy was expressed as the percentage of tumor growth inhibition (%TGI), calculated according to the formula: %TGI = 100 − (RTV_T_/RTV_C_ × 100); where RTV_T_ is the mean relative tumor volume of treated mice and RTV_C_ is the mean relative tumor volume of untreated control mice determined at Day 18, when the first control mouse was euthanized. Tumor growth delay indices (TGDI) were calculated according to the formula: TGDI = TGD_T_/TGD_C_. This was defined as the mean tumor growth delay ratio of treated (TGD_T_) and untreated animals (TGD_C_) which was required to increase the RTV 2- and 5-fold, as previously reported [25,31]. The data sets were analyzed for significance using a one-way ANOVA with Tukey’s multiple comparison post-test using GraphPad Prism software (version 7). A *p*-value of <0.05 was considered as statistically significant.

### 2.9. Determination of Blood Plasma Parameters

Blood was sampled from the retrobulbar vein under isoflurane inhalation anesthesia prior to euthanasia of the mice when they had reached an endpoint criterion. Blood samples were centrifuged (4 °C, 20 min, 1600 rpm) and the supernatant (blood plasma) was analyzed using a Fuji Dri-Chem 4000i analyzer (Polymed Medical Center AG, Glattbrugg, Switzerland) measuring creatinine (CRE), blood urea nitrogen (BUN), alkaline phosphatase (ALP), and total bilirubin (TBIL). Data were analyzed for significance using a one-way ANOVA test (GraphPad Prism software, version 7.0). A *p*-value of <0.05 was considered as statistically significant.

### 2.10. Histopathological Investigations

Pathological evaluation of mice without tumors was conducted two weeks after injection of ^47^Sc-folate, ^177^Lu-folate, and ^90^Y-folate, respectively, at two different activity levels. A full macroscopic examination was performed in each animal and the kidneys, bone marrow (sternum and femur), and spleen were sampled for histological assessment. The tissues were fixed in 4% neutral-buffered formalin and routinely embedded in paraffin wax. Before paraffin embedding, sternum and femur tissues were decalcified at room temperature in an ethylene- diamine-tetraacetic acid (EDTA)-citrate solution for 2 days. Sections of 3–5 µm thickness were prepared on glass slides and routinely stained with hematoxylin eosin (HE). Histological lesions were semi-quantitatively scored by a veterinary pathologist in a blind manner using a severity grading scheme that ranged from 0 to 5. This indicated no lesions (score 0), minimal (score 1), mild (score 2), moderate (score 3), moderate to severe (score 4) and severe damage (score 5). Renal microscopic changes of the glomerular, tubular, and interstitial compartments were scored separately and the sum of the three values used to obtain a cumulative score representing radiation nephropathy damage, as described previously [32]. Histological evaluation of the spleen was conducted to determine radiation injury in the lymphoid cells of the white pulp using a score of 0 (no lymphoid depletion) to a score of 5 (severe lymphoid depletion), as well as the extra-medullary hematopoiesis (EMH) of the red pulp scoring from a score of 0 (no EMH observed) to a score of 5 (large numbers of EMH precursors observed). The hematopoietic function was also evaluated in the bone marrow by estimating the overall cellularity using a score of 0 (no reduction in cellularity) to a score of 5 (severe reduction in cellularity) and the proportion of the different cellular lineages (including granulocytic precursors, erythroid cells and megakaryocytes).

## 3. Results

### 3.1. Radiofolate Preparation and Tumor Cell Internalization Studies

The ^47^Sc was obtained in dilute hydrochloric acid in sufficient quality and quantity for the required radiolabeling and subsequent preclinical study (Supplementary Materials, Figure S1). For the in vitro studies reported herein, the radiolabeling of the folate conjugate with ^47^Sc, ^177^Lu, and ^90^Y was performed at molar activities up to 12.5 MBq/nmol, 20 MBq/nmol, and 10 MBq/nmol, respectively, yielding the product with a chemical purity >97% (Supplementary Materials, Figure S2).

**Figure 1:**
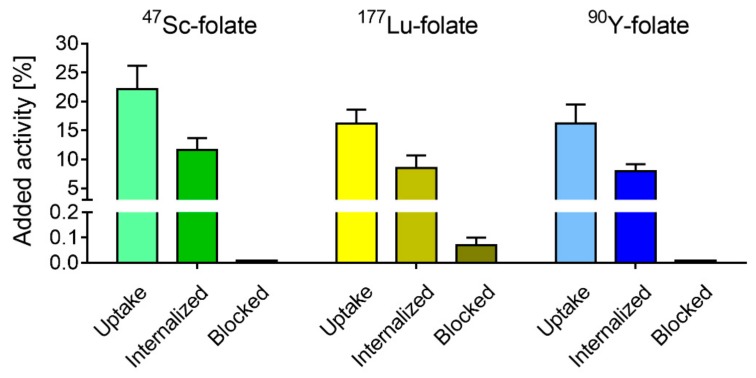
IGROV-1 tumor cell uptake and internalization of ^47^Sc-folate, ^177^Lu-folate, and ^90^Y-folate after 4 h incubation. Blockade of the folate receptor (FR) by co-incubation of the IGROV-1 tumor cells with folic acid resulted in less than 0.1% uptake. The graph represents the average ± SD of three or four independent experiments performed in triplicate.

Cell uptake and internalization were studied using IGROV-1 tumor cells in culture. The total uptake (surface-exposed FR-bound and internalized fraction) of all radiofolates after 4 h incubation was in the range 17−21% of added activity, while the internalized fraction was 7−12%. The uptake in IGROV-1 tumor cells which were incubated with excess folic acid was <0.1%, indicating the FR-dependent uptake of the radiofolates (Figure 1).

### 3.2. Cell Viability Assay

**Figure 2:**
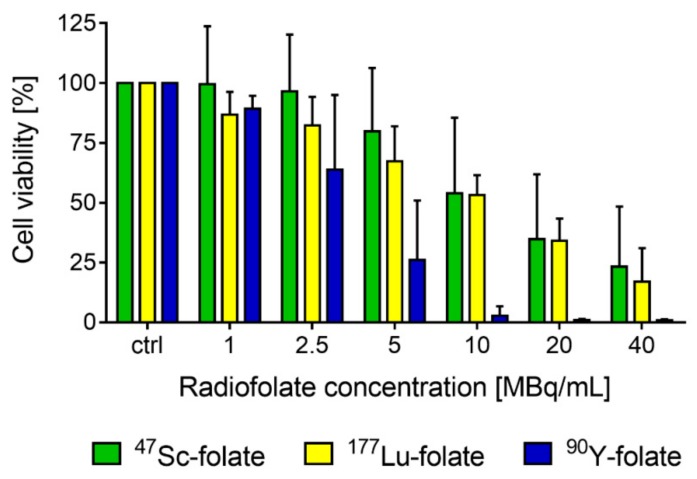
IGROV-1 cell viability after treatment with different quantities of ^47^Sc-folate (green), ^177^Lu-folate (yellow), and ^90^Y-folate (blue). The average of four, five, or three independent experiments is shown for ^47^Sc-folate, ^177^Lu-folate, and ^90^Y-folate, respectively.

The viability of the IGROV-1 tumor cells was reduced after treatment with the folate conjugate labeled with each radionuclide. A concentration of 5 MBq/mL reduced the viability to 80% and 67% in the case of ^47^Sc-folate and ^177^Lu-folate, respectively (Figure 2). Treatment of the tumor cells with the same activity concentrations of ^90^Y-folate reduced the viability of the cells to 26%. A radiofolate concentration of 20 MBq/mL reduced the viability of cells to ~35% and ~34% in the case of ^47^Sc-folate and ^177^Lu-folate, respectively, and caused almost complete cell killing with <1% viable cells when using ^90^Y-folate.

### 3.3. Biodistribution and Dosimetry

The mean absorbed dose to the tumor and kidneys was calculated by taking the physical properties of the radionuclide into account, along with the size of the tissue, and the uptake, residence, and clearance of the radioactivity over time. For this purpose, biodistribution studies were performed in IGROV-1 tumor-bearing mice using ^177^Lu-folate as a surrogate for all radiofolates, based on the assumption that the tissue distribution profile of all radiofolates would be equal—as previously shown for ^44^Sc/^47^Sc-labeled tumor targeting ligands and their ^177^Lu-labeled counterparts [14,33]. The biodistribution data of ^177^Lu-folate in IGROV-1 tumor-bearing mice are available in the Supplementary Materials, Tables S1 and S2. The mean tumor absorbed doses of ^47^Sc-folate, ^177^Lu-folate, and ^90^Y-folate were calculated as 1.7 Gy/MBq, 2.1 Gy/MBq, and 4.3 Gy/MBq, respectively, and calculations for the mean absorbed kidney doses revealed 1.8 Gy/MBq, 2.3 Gy/MBq, and 4.4 Gy/MBq, respectively. In order to obtain a mean absorbed tumor dose of ~21 Gy, mice had to be treated with 12.5 MBq, 10 MBq, and 5 MBq of ^47^Sc-folate, ^177^Lu-folate, and ^90^Y-folate, respectively (Supplementary Materials, Table S3). This quantity of injected activity resulted in a mean absorbed kidney dose of ~22−23 Gy.

### 3.4. Therapy Experiments

The therapeutic effect of ^47^Sc-folate, ^177^Lu-folate, and ^90^Y-folate was assessed in IGROV-1 tumor-bearing mice over a period of eight weeks. The quantity of applied radiofolate was adapted for each radionuclide, in order to obtain comparable mean absorbed doses to tumor xenografts and kidneys.

**Figure 3:**
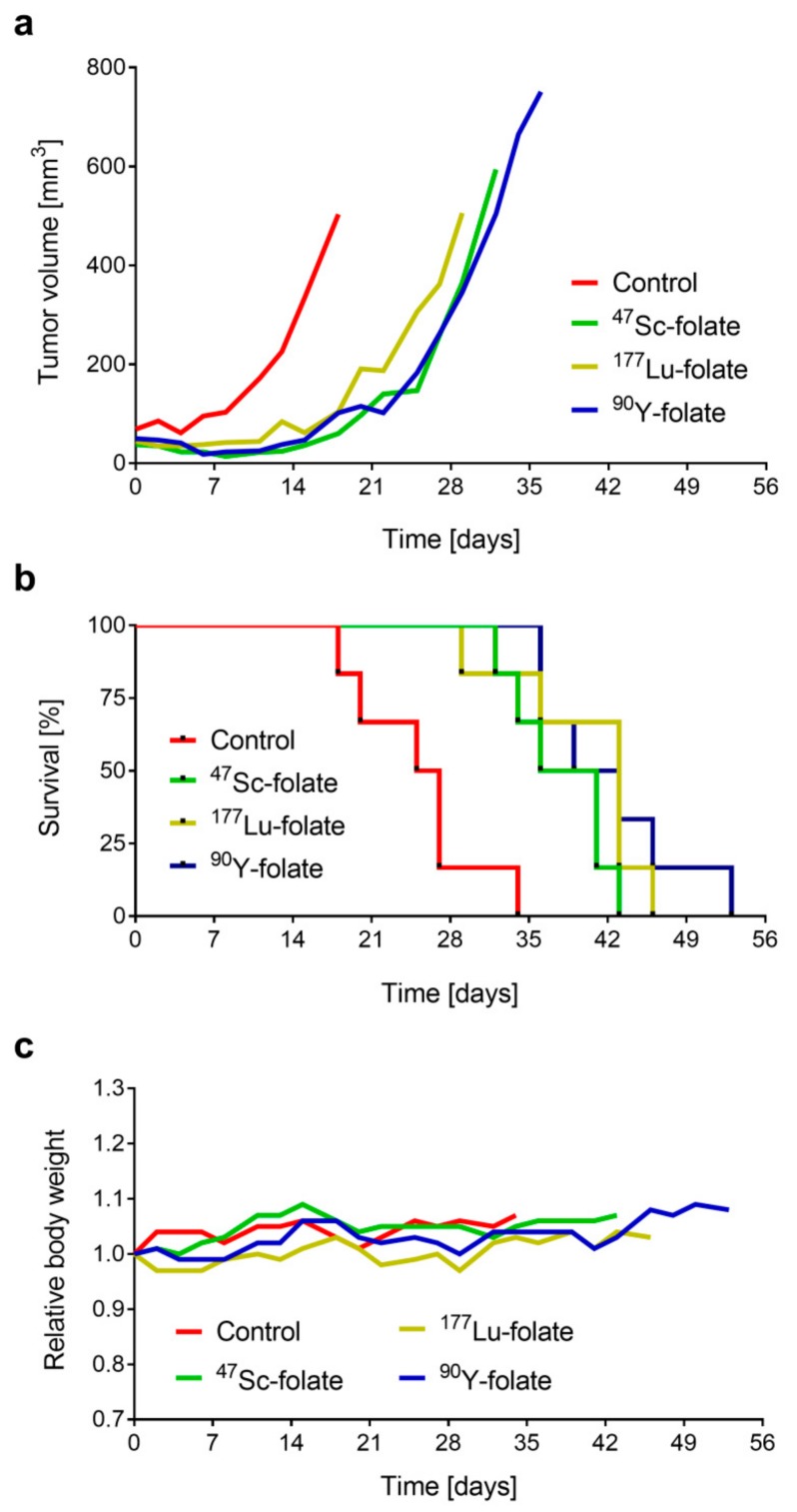
Results of the therapy study of mice which received ~21 Gy tumor dose. (**a**) Tumor growth curves indicated as the average of IGROV-1 tumor sizes in mice treated with ^47^Sc-, ^177^Lu-, and ^90^Y-folate, respectively. (**b**) Survival curves of mice treated with ^47^Sc-, ^177^Lu-, and ^90^Y-folate resulting in median survival times of 39, 43, and 41 days, respectively. (**c**) Relative body weight of mice from each group over the period of investigation.

In all treated groups, the average tumor growth was delayed in comparison to untreated control mice (Figure 3a; Supplementary Materials, Figure S3). The tumor growth inhibition (TGI) in treated mice was all in the same range of 67% to 76%, irrespective of whether the mice received ^47^Sc-folate, ^177^Lu-folate, or ^90^Y-folate, respectively (*p* > 0.05). As an additional measure of the therapeutic effect, tumor growth delay indices (TGDI) were calculated. The TGDI_2_, defined as the mean tumor growth delay ratio of treated and untreated mice required to double the relative tumor volume (RTV), was in the range 1.5–1.7. The TGDI calculated for the tumor volumes to increase 5-fold was highest in mice that received ^90^Y-folate (1.6), followed by ^177^Lu-folate (1.5), and ^47^Sc-folate (1.3). These values revealed a significantly increased TGDI_2_ and TGDI_5_ for the treated groups as compared to untreated controls (*p* < 0.05); however, no statistically significant difference was found among the radiofolate-treated groups (*p* > 0.05) (Table 2).

The survival curves reflected the results of tumor growth, as the endpoint criterion was always due to oversized tumors (Figure 3b). The median survival of mice in each group ranged from 39 to 43 days in treated animals, which was significantly enhanced as compared to 26 days median survival in control mice. The average relative body weight (RBW) was stable in all groups, indicating that the treatment did not have a negative influence on the overall condition of the mice (Figure 3c). There was one case in the ^177^Lu-folate-treated group that experienced more than 10% body weight loss, which can be ascribed to the fast tumor growth which already required euthanasia at Day 29 after treatment (Supplementary Materials, Figure S4).

On the day of euthanasia, creatinine (CRE) levels were below the detection limit in all cases. Blood urea nitrogen (BUN), alkaline phosphatase (ALP), and total bilirubin (TBIL) levels were comparable among treated mice and untreated controls, irrespective of the radiofolate that was applied (Supplementary Materials, Table S4).

### 3.5. Histopathological Assessment

The assessment of the kidneys, bone marrow, and spleen was performed two weeks after injection of the radiofolates, applied at the same activities as in the therapy study, as well as at the doubled quantity of the respective radiofolate (Supplementary Materials, Tables S5 and S6). The histological examination of the renal tissue did not show evidence of radiation nephropathy in any of the groups treated with low activity. This was also the case for the majority of animals treated with high activity (Supplementary Materials, Figure S5). Only two animals treated with 25 MBq ^47^Sc-folate exhibited occasional glomeruli with mild reduction of the mesangial capillaries; however, no other alterations in the tubular and interstitial compartments were detected, and the overall renal cortex did not show signs of parenchymal shrinkage.

**Figure 4:**
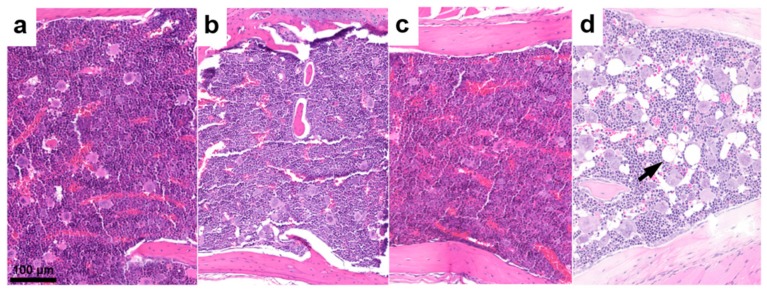
Representative histological findings in the bone marrow (sternum) of mice euthanized two weeks after injection of the radiofolates. (**a**) Tissue of a control mouse that received saline; (**b**) tissue of a mouse treated with 25 MBq ^47^Sc-folate; (**c**) tissue of a mouse treated with 20 MBq ^177^Lu-folate; (**d**) tissue of a mouse that received 10 MBq ^90^Y-folate. The number of hematopoietic cells is reduced and replaced by adipocytes (arrow).

Control animals and mice injected with lower activity showed adequate hematopoietic cellularity in the bone marrow, with normal proportions of erythroid cells, granulocytic precursors, and megakaryocytes (Figure 4a). The hematopoietic cellularity was also unremarkable in all the animals treated with high activity (Figure 4b,c), apart from two animals that received higher activity of ^90^Y-folate (10 MBq), which showed mild reduction of the bone marrow cellularity accompanied by adipocyte replacement (Figure 4d). Hematopoietic cell activity was also evaluated in the red pulp of the spleen by determining the presence of extra-medullary hematopoiesis (EMH). The overall cellularity of EMH varied between animals and groups; however, mice treated with 10 MBq ^90^Y-folate tended to have lower values compared to the other groups. On the other hand, none of the animals showed evidence of lymphocyte damage in the white pulp of the spleen (data not shown).

## 4. Discussion

^47^Sc has been previously proposed as a potential therapeutic match to ^44^Sc, a PET nuclide with great potential for clinical translation [14,19,20,33]. Preclinical studies to investigate ^47^Sc for therapeutic application have been scarce [14], however, and a clinical application does not yet exist due to the challenging production routes of this novel radionuclide [12,13,34,35,36]. In the present study, the aim was to investigate ^47^Sc in a preclinical setting and compare its effect with ^177^Lu and ^90^Y, two established therapeutic nuclides which have been extensively employed in clinics over the last two decades.

The production of ^47^Sc was performed via the ^46^Ca(n,γ)^47^Ca→^47^Sc nuclear reaction as previously reported [12]; however, the ^46^CaO used in this work provides better chemical stability during irradiation, which allows the irradiation of higher Ca-target masses. With the transfer into another chemical form, no additional impurities were introduced, hence, identical post-irradiation γ-spectra were obtained, as previously reported [12].

As expected, similar results were found in the uptake and internalization of ^47^Sc-folate, ^177^Lu-folate, and ^90^Y-folates in ovarian IGROV-1 cancer cells. The data were in line with previously-reported results, where ^47^Sc-folate was investigated with KB tumor cells [14]. Cell viability experiments, performed with increasing quantities of ^47^Sc-folate, ^177^Lu-folate, and ^90^Y-folate, resulted in different effects, dependent on the decay characteristics of the radionuclide employed. The most prominent decrease in viability was found when using ^90^Y-folate, which was in line with the fact that ^90^Y emits β^−^-particles of the highest energy. ^47^Sc-folate and ^177^Lu-folate showed similar effects on IGROV-1 tumor cells.

Dose calculations revealed that to obtain the same mean absorbed dose to the tumor and kidneys, slightly higher quantities of ^47^Sc-folate than of ^177^Lu-folate were necessary due to the different half-lives of ^47^Sc and ^177^Lu. The high β^−^-energy of ^90^Y was responsible for an increased dose deposition, which required only half of the activity of ^90^Y-folate to obtain the same mean absorbed dose as for ^47^Sc-folate and ^177^Lu-folate. Treatment of IGROV-1 tumor-bearing mice with these adapted activities had similar effects irrespective of whether ^47^Sc-folate, ^177^Lu-folate, or ^90^Y-folate was used. Consistent with these findings, the median survival time of treated mice was comparable in each group. These results were also comparable to those obtained previously in KB tumor-bearing mice, where 10 MBq ^47^Sc-folate resulted in an equal median survival time of 38.5 days [14] as for the IGROV-1 tumor mouse model when treated with 12.5 MBq ^47^Sc-folate (median survival: 39 days).

Due to the higher energy of ^90^Y and, as a consequence, an increased tissue range of the emitted β^−^-particles in comparison to the β^−^-particles emitted by ^177^Lu, ^90^Y-folate may have the disadvantage of more side effects to kidneys as observed with somatostatin analogues (^90^Y-DOTATOC/^90^Y-DOTATATE) in clinical studies [8,9,37]. Based on the more similar mean β^−^-energies of ^47^Sc and ^177^Lu, kidney damage would most likely be observed at the same dose levels for ^47^Sc- and ^177^Lu-labeled tumor targeting agents. In this study, only sporadic and very mild glomerular changes were observed, and none of the mice experienced severe renal side effects that would have been detectable by histopathological investigations. It has to be critically acknowledged, however, that radionephrotoxicity is not commonly observed at short time points after injection but rather, several months after radionuclide therapy [38]. It is, therefore, very likely that the higher activity levels of the applied radiofolates, resulting in a kidney dose of 42–44 Gy, would manifest as signs of radiotoxicity six to eight months after treatment.

Severe side effects were not observed in other tissues and organs. Only mice treated with 10 MBq ^90^Y-folate showed mildly reduced extra-medullary hematopoiesis in the spleen, and two animals also exhibited reduced cellularity in the bone marrow, which could be indicative of irradiation-related damage. The amount of extra-medullary hematopoiesis varied between animals and groups, which means that these changes could represent a transient condition rather than a direct radiotoxic event. Since later time points were not investigated in this study, it remains unknown whether hematopoietic changes would recover or, instead, progress to a more severe stage.

As expected, the application of ^47^Sc-folate revealed similar anti-tumor effects as that for treatment using its ^177^Lu- and ^90^Y-labeled counterparts when the applied activity was adjusted to obtain the same mean absorbed tumor dose. In this mouse model, differences among the single therapy groups were not determined, indicating that ^47^Sc may be used under the same medical circumstances as ^177^Lu and ^90^Y, respectively. The relatively short half-life of ^47^Sc makes its use particularly attractive in combination with fast-clearing tumor targeting ligands, including peptides and small molecules.

It has to be critically acknowledged that a potential advantage of ^47^Sc over ^177^Lu remains questionable, particularly due to the higher intensity of γ-emission (^47^Sc: Eγ = 159 keV; I = 68%) which may unnecessarily contribute to the patient dose. On the other hand, this characteristic may be advantageous in cases where an exact dosimetry is required, as recently claimed for ^67^Cu (oral communication), which has very similar decay characteristics to ^47^Sc with regard to half-life, β^−^-particle emission, and intensity of γ-ray emission. Should it be the case, ^47^Sc would probably be preferred over ^67^Cu, due to its easier coordination chemistry and, hence, the opportunity of using it with any tumor-targeting agent that comprises a DOTA chelator.

## 5. Conclusions

The in vitro and in vivo evaluation of ^47^Sc indicated comparable efficacy in tumor therapy to that of ^177^Lu and ^90^Y, when applied at activities that result in the same mean absorbed tumor doses. These findings confirm the accuracy of the dose estimation to tumors, which was performed for ^47^Sc-folate, ^177^Lu-folate, and ^90^Y-folate, respectively. Overall, the characteristics of ^47^Sc were comparable to those of ^177^Lu and, hence, these two radionuclides may be interchangeable. It has to be critically acknowledged, however, that the production of ^47^Sc is costly and, hence, the radionuclide is not currently available in large quantities that would enable clinical translation.

^47^Sc could be advantageous in combination with molecules of fast pharmacokinetics, where a shorter half-life would be desirable, and in situations where an intense γ-emission was of interest. Certainly, ^47^Sc could play a pivotal role for radiotheragnostic application in tandem with ^43^Sc/^44^Sc, enabling the preparation of chemically-identical radiopharmaceuticals for PET imaging and therapeutic applications. This will be feasible as soon as the production of the Sc radioisotope family has been fully established to make them readily available in large quantities, at high quality, and at a reasonable cost.

## Figures and Tables

**Table 1:** Decay properties ^1^ of the therapeutic radionuclides, ^47^Sc, ^177^Lu, and ^90^Y.

Radionuclide	Half-Life	Eβ^−^ Average	Eγ (Intensity) ^2^	Availability
^47^Sc	3.35 d	162 keV	159 keV (68%)	produced as part of this research project
^177^Lu	6.65 d	134 keV	113 keV (6%) 208 keV (10%)	commercially available
^90^Y	2.67 d	934 keV	none	commercially available

^1^https://www.nndc.bnl.gov/nudat2/; ^2^ γ-Lines relevant for SPECT imaging.

**Table 2:** Analysis of the therapy experiment.

Radioligand	Activity [MBq]	Tumor D [Gy]	Kidney Dose [Gy]	Euthanasia of the First Mouse	Median Survival [days]	TGI [%]	TGDI_2_	TGDI_5_
Saline	-	-		Day 18	26	-	1.0 ± 0.3	1.0 ± 0.2
^47^Sc-folate	12.5	21.3	22.5	Day 32	39	69 ± 17 ^1^	1.5 ± 0.3 ^1^	1.3 ± 0.2 ^1^
^177^Lu-folate	10	20.8	23.0	Day 29	43	67 ± 28 ^1^	1.5 ± 0.5 ^1^	1.5 ± 0.3 ^1^
^90^Y-folate	5	21.5	22.0	Day 36	41	76 ± 15 ^1^	1.7 ± 0.2 ^1^	1.6 ± 0.1 ^1^

^1^ values of treated mice were significantly different from values of untreated controls (*p* < 0.05), but no statistically significant difference was shown among the treated groups (*p* > 0.05).

## Supplementary Materials

The following are available online at https://www.mdpi.com/1999-4923/11/8/424/s1, Details of the separation of ^47^Sc from the target material (including Figure S1); Radiolabeling and quality control of the radioconjugates (including Figure S2); Cell internalization; Biodistribution studies (including Tables S1 and S2); Dosimetry estimation (including Table S3); Therapy experiments (including Figures S3 and S4 and Table S4); Toxicity Assessment (including Tables S5 and S6 and Figure S5).
