# Supplementary material for: Therapeutic Potential of 47Sc in Comparison to 177Lu and 90Y: Preclinical Investigations
Source: Pharmaceutics. 2019 Aug 20;11(8):424. doi: 10.3390/pharmaceutics11080424 (PMC6723926; doi:10.3390/pharmaceutics11080424)
Supplement: Supplementary file 1 [file pharmaceutics-11-00424-s001.pdf]

*pharmaceutics*

# Supplementary Materials: Therapeutic Potential of $^{47}Sc$ in Comparison to $^{177}Lu$ and $^{90}Y$: Preclinical Investigations

**Klaudia Siwowska, Patrycja Guzik, Katharina A. Domnanich, Josep M. Monné Rodríguez, Peter Bernhardt, Bernard Ponsard, Roger Hasler, Francesca Borgna, Roger Schibli, Ulli Köster, Nicholas P. van der Meulen and Cristina Müller**

## 1. Separation of $^{47}Sc$ from the Target Material

**Purpose:** Scandium-47 ($^{47}Sc$) was produced using the $^{46}Ca(n,\gamma)^{47}Ca \rightarrow ^{47}Sc$ nuclear reaction as previously reported [1,2], however, in the course of these studies, the production and separation processes were optimized to use $^{46}CaO$ target material instead of $^{46}Ca(NO_3)_2$.

**Target preparation:** Targets were prepared as previously reported [2], with the additional step of converting the $^{46}Ca(NO_3)_2$ into $^{46}CaO$ at 600°C for 30 minutes (Figure S1). A transformation into this chemically stable form of target material was required, as more Ca could be irradiated, as well as the fact that the oxide is a more stable compound under irradiation conditions.

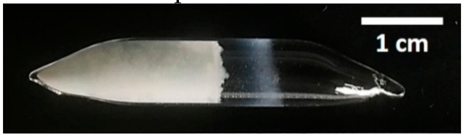


**Figure S1.** Representative picture of an ampoule containing enriched $^{46}CaO$ target material used for $^{47}Sc$ production.

**Separation Method:** The $^{46}Ca$ ampoules were irradiated at the relevant research reactor facility and, subsequently, transported to PSI. The irradiated target material was dissolved in 1.0 M $HNO_3$ and loaded onto a DGA resin column. Subsequent rinsing with 0.1 M $HNO_3$ enabled the removal of stable $^{46}Ca$ and radioactive $^{47}Ca$. The waste solutions of the steps involving $HNO_3$ solution were collected in a separate container, to allow the radioactive $^{47}Ca$ ($T_{1/2}$ = 4.5 d) to decay to $^{47}Sc$ and subject this solution to two subsequent separations, every 3 days after the previous separation. Afterwards, the column was rinsed with 3.0 M HCl, in order to convert the resin to the chloride form and to rid the product of impurities, respectively. The sorbed $^{47}Sc$ was eluted from the DGA resin with 0.1 M HCl and acidified with 6.0 M HCl to yield a 3.0 M HCl solution. The solution was loaded to a smaller DGA resin column and the $^{47}Sc$ eluted by fractionized collection using 0.05 M HCl, as previously reported [2].

**Results:** The $^{47}Sc$ final product featured a high radionuclidic purity, containing <0.005% $^{46}Sc$ upon initial separation and no $^{46}Sc$ whatsoever in subsequent separations. The product rendered a reproducible radiolabeling of the folate conjugate, possible up to a molar activity of 12.5 MBq/nmol.

## 2. Radiolabeling and Quality Control of the Radioconjugates

**Purpose:** Radiolabeling of the folate conjugate with $^{47}Sc$, $^{177}Lu$ and $^{90}Y$ was performed under standard labeling conditions, followed by quality control using HPLC.

**Methods:** Radiolabeling of the radiofolate with $^{47}Sc$, $^{177}Lu$ and $^{90}Y$ was carried out at a molar activity up to 12.5 MBq/nmol, 20 MBq/nmol and 10 MBq/nmol, respectively. $^{47}ScCl_3$, $^{177}LuCl_3$ in 0.05 M HCl or $^{90}YCl_3$ in 0.04 M HCl were mixed with sodium acetate (0.5 M, pH 8) in a 5:1 ratio to obtain a solution of pH ~4.5. After the addition of the folate conjugate (1 mM stock solution), the reaction vial was incubated for 10-15 min at 95 °C.

Quality control of the radiofolates ($^{47}Sc$-folate, $^{177}Lu$-folate and $^{90}Y$-folate) was performed with a Merck Hitachi LaChrom HPLC system, equipped with a D-7000 interface, a L-7200 autosampler, a radioactivity detector (LB 506 B from Berthold) and a L-7100 pump connected with a reversed-phase C18 column (5 µm, 150 × 4.6 mm, Xterra™, MS, Waters, USA). The mobile phase consisted of 0.1%

TFA in MilliQ water (A) and ACN (B) using a linear gradient of solvent A (95–20% over 15 min) in solvent B at a flow rate of 1 mL/min. An aliquot (~0.3 MBq) of the radiolabeling solution was diluted in 100 μL MilliQ water containing sodium diethylenetriamine pentaacetic acid (Na-DTPA; 50 μM) for analysis.

**Results:** Representative chromatograms of $^{47}$Sc-folate, $^{177}$Lu-folate and $^{90}$Y-folate are shown in Figure S2. No difference in the retention time ($t_R$ = 11.4 ± 0.1 min) was observed among the radiofolates. All radiofolates were obtained at a radiochemical purity ≥97% and were used for in vitro and in vivo evaluation without further purification.

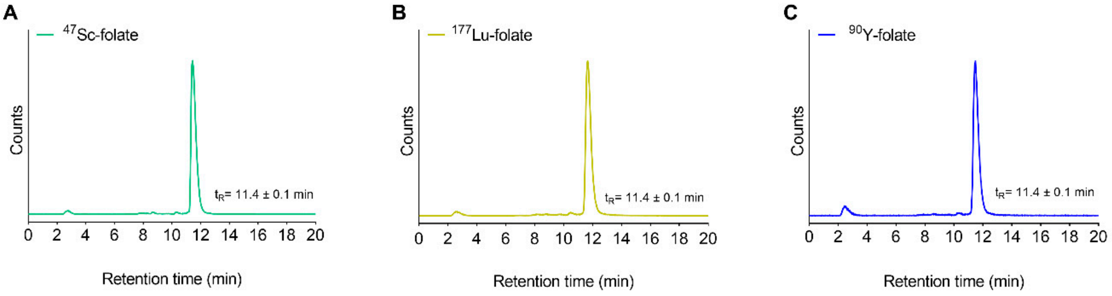


**Figure S2.** Representative chromatograms obtained for quality control of all radiofolates; (**a**) $^{47}$Sc-folate, (**b**) $^{177}$Lu-folate and (**c**) $^{90}$Y-folate. The retention time presents the average ± SD of $n$ = 4 chromatograms.

## 3. Cell Internalization Experiments

**Purpose:** Cell experiments were performed to demonstrate FR-specific uptake and internalization of the folate conjugate labeled with the radionuclides in question ($^{47}$Sc, $^{177}$Lu and $^{90}$Y) using FR-positive IGROV-1 ovarian cancer cells.

**Methods:** IGROV-1 tumor cells were seeded in 12-well plates (0.5 × 10$^6$ cells in 2 mL FFRPMI medium/well) allowing cell adhesion and growth overnight at 37 °C, 5% $CO_2$. After removal of the supernatant, the cells were washed once with PBS prior to the addition of FFRPMI medium without supplements (975 μL/well). $^{47}$Sc-folate, $^{177}$Lu-folate or $^{90}$Y-folate were labeled at a molar activity of 10 MBq/nmol. The radiofolates (25 μL, ~38 kBq, ~3.75 pmol) were added to each well. In some cases, cells were incubated with excess folic acid (100 μM) to block FRs on the surface of the cancer cells. After incubation of the well plates for 4 h at 37 °C, the cells were washed with ice-cold PBS to determine total uptake of $^{47}$Sc-folate, $^{177}$Lu-folate and $^{90}$Y-folate. In order to assess the fraction of internalized radiolabeled folates, the cells were additionally washed with a stripping buffer (aqueous solution of 0.1 M acetic acid and 0.15 M NaCl, pH 3 [3]) to release FR-bound radiofolates from the cell surface. The tumor cells were lysed by addition of NaOH (1.0 M, 1 mL) to each well, followed by transfer of the cell suspensions to tubes for measuring in a γ-counter (Perkin Elmer, Wallac Wizard 1480). The results were expressed as percentage of total added activity and graphs were prepared using GraphPad Prism software (version 7.0).

**Results:** The results are reported in the main article (Figure 1).

## 4. Biodistribution Study

**Purpose:** Time-dependent biodistribution experiments were performed with $^{177}$Lu-folate in IGROV-1 tumor-bearing mice in order to be able to estimate the mean absorbed dose. It was assumed that the distribution profile of $^{47}$Sc-folate and $^{90}$Y-folate would be equal to the distribution of $^{177}$Lu-folate.

**Methods:** Biodistribution studies were performed as previously reported by Siwowska et al. [4].

**Results:** Biodistribution data obtained at 1 h, 4 h, 24 h, 48 h, 4 d, 5 d, 7 d and 10 d after administration of $^{177}$Lu-folate (as a representative for all radioconjugates), using IGROV-1 tumor-bearing mice, are listed in Tables S1/S2. The tumor uptake increased from 29 ± 3% IA/g at 1 h p.i. to 33 ± 9% IA/g at 4 h p.i. and reached the highest value 24 h after injection of the radiofolate (39 ± 8% IA/g) (Table S1). The accumulation of the activity in the tumor began to drop at 48 h p.i. and decreased

to 3.5 ± 1.8% IA/g at the last investigated time point, 10 days after injection (Table S2). Kidney accumulation was as prominent as for the tumor and increased up to 24 h after injection, when it reached 35 ± 10% IA/g. Nevertheless, the highest tumor-to-kidney ratio of accumulated activity was observed after 4 h (1.2 ± 0.5). In the blood, radioactivity was present at significant amounts until 4 h p.i. and dropped to 0.82 ± 0.16% IA/g at 24 h p.i.. Radioactivity was also detected in salivary glands, lung and liver, however, it decreased rapidly over time.

**Table S1.** Biodistribution 1, 4, 24 and 48 h after injection of $^{177}$Lu-folate conjugate in IGROV-1 tumor-bearing female nude mice. The values are presented as percentage of injected activity per gram tissue [% IA/g].

| | **1 h p.i.** | **4 h p.i.** | **24 h p.i.** | **48 h p.i.** |
|---|---|---|---|---|
| Blood | 16 ± 3 | 6.7 ± 0.7 | 0.82 ± 0.16 | 0.50 ± 0.15 |
| Lung | 6.7 ± 1.2 | 4.8 ± 0.4 | 1.6 ± 0.3 | 0.71 ± 0.29 |
| Spleen | 2.7 ± 0.2 | 1.3 ± 0.1 | 0.63 ± 0.07 | 0.53 ± 0.08 |
| Kidneys | 21 ± 3 | 29 ± 6.1 | 35 ± 10 | 17 ± 3 |
| Stomach | 1.7 ± 0.7 | 1.1 ± 0.2 | 0.63 ± 0.16 | 0.34 ± 0.04 |
| Intestines | 1.6 ± 0.2 | 0.9 ± 0.3 | 0.22 ± 0.05 | 0.23 ± 0.07 |
| Liver | 3.5 ± 0.4 | 2.7 ± 0.3 | 2.3 ± 0.6 | 1.3 ± 0.1 |
| Salivary glands | 8.6 ± 2.0 | 9.9 ± 2.7 | 7.2 ± 2.5 | 2.4 ± 0.2 |
| Muscle | 1.6 ± 0.1 | 1.4 ± 0.3 | 1.0 ± 0.4 | 0.72 ± 0.10 |
| Bone | 2.0 ± 0.2 | 1.4 ± 0.1 | 0.88 ± 0.23 | 0.51 ± 0.04 |
| IGROV-1 tumor | 29 ± 3 | 33 ± 9 | 39 ± 8 | 15 ± 3 |
| Tumor-to-blood | 1.3 ± 0.1 | 5.0 ± 1.8 | 47 ± 7 | 30 ± 6 |
| Tumor-to-liver | 4.4 ± 1.0 | 7.5 ± 4.4 | 12 ± 9 | 7.2 ± 5.5 |
| Tumor-to-kidney | 1.0 ± 0.0 | 1.2 ± 0.5 | 1.1 ± 0.2 | 0.86 ± 0.02 |

Values shown represent the mean ± S.D. of data from three animals (*n* = 3).

**Table S2.** Biodistribution 4, 5, 7 and 10 days after injection of $^{177}$Lu-folate conjugate in IGROV-1 tumor-bearing female nude mice. The values are presented as percentage of injected activity per gram tissue [% IA/g].

| | **4 d p.i.** | **5 d p.i.** | **7 d p.i.** | **10 d p.i.** |
|---|---|---|---|---|
| Blood | 0.15 ± 0.06 | 0.11 ± 0.02 | 0.03 ± 0.01 | 0.01 ± 0.00 |
| Lung | 0.54 ± 0.10 | 0.40 ± 0.07 | 0.18 ± 0.02 | 0.11 ± 0.01 |
| Spleen | 0.42 ± 0.10 | 0.50 ± 0.03 | 0.37 ± 0.01 | 0.20 ± 0.01 |
| Kidneys | 16 ± 6 | 16 ± 3 | 8.7 ± 1.1 | 6.3 ± 1.2 |
| Stomach | 0.23 ± 0.05 | 0.25 ± 0.07 | 0.09 ± 0.03 | 0.06 ± 0.02 |
| Intestines | 0.20 ± 0.12 | 0.14 ± 0.02 | 0.07 ± 0.02 | 0.03 ± 0.01 |
| Liver | 0.83 ± 0.36 | 0.60 ± 0.09 | 0.32 ± 0.05 | 0.24 ± 0.02 |
| Salivary glands | 1.9 ± 0.2 | 1.7 ± 0.3 | 1.1 ± 0.5 | 0.56 ± 0.10 |
| Muscle | 0.52 ± 0.12 | 0.27 ± 0.08 | 0.18 ± 0.13 | 0.06 ± 0.01 |
| Bone | 0.27 ± 0.06 | 0.32 ± 0.05 | 0.14 ± 0.05 | 0.10 ± 0.02 |
| IGROV-1 tumor | 13 ± 6 | 13 ± 4 | 7.1 ± 2.4 | 3.5 ± 1.8 |
| Tumor-to-blood | 86± 17 | 121 ± 49 | 246 ± 116 | 309 ± 28 |
| Tumor-to-liver | 9.8 ± 8.2 | 14 ± 13 | 12 ± 10 | 11 ± 9.2 |
| Tumor-to-kidney | 0.81 ± 0.15 | 0.77 ± 0.09 | 0.84 ± 0.33 | 0.59 ± 0.25 |

Values shown represent the mean ± S.D. of data from three animals (*n* = 3).

## 5. Dosimetry Estimation

**Purpose:** Dosimetry calculations were performed in order to estimate the quantity of activity of $^{47}$Sc-folate, $^{177}$Lu-folate or $^{90}$Y-folate, respectively, which had to be injected to obtain the same mean absorbed dose to tumors and kidneys.

**Methods:** The dosimetry method is described in the main article.

**Results:** A mono-exponential fit was applied to the biokinetic data. The time integrated activity concentration for $^{47}$Sc, $^{177}$Lu and $^{90}$Y in the tumor were equal to $7.3 \times 10^4$, $9.4 \times 10^4$ and $6.5 \times 10^4$ decay/g,

respectively. The time-integrated activity concentration for $^{47}$Sc, $^{177}$Lu and $^{90}$Y in the kidneys were equal to 7.6 × 10$^4$, 10 × 10$^4$ and 6.6 × 10$^4$ decay/g, respectively. The absorbed electron energy fractions for the tumors were 0.89, 0.93 and 0.44 for $^{47}$Sc, $^{177}$Lu and $^{90}$Y, respectively. The absorbed electron energy fractions for the kidneys were 0.91, 0.95 and 0.45 for $^{47}$Sc, $^{177}$Lu and $^{90}$Y, respectively. The specific mean absorbed doses are presented in the main article.

To obtain the dose of ~21 Gy in the tumors, mice had to be treated with 12.5 MBq, 10 MBq and 5 MBq of $^{47}$Sc-folate, $^{177}$Lu-folate and $^{90}$Y-folate, respectively (Table S3). This quantity of injected activity resulted in a mean absorbed dose of ~22–23 Gy to the kidneys, which should – according to previously published data [5]—not cause radionephrotoxicity.

**Table S3.** Mean absorbed dose in tumors and kidneys of IGROV-1 tumor-bearing mice treated with $^{47}$Sc-, $^{177}$Lu- and $^{90}$Y-folate in the therapy study.

| Compound | Quantity [MBq] | Tumor Dose [Gy] | Kidney Dose [Gy] |
|---|---|---|---|
| saline | - | - | - |
| $^{47}$Sc-folate | 12.5 | 21.3 | 22.5 |
| $^{177}$Lu-folate | 10 | 20.8 | 23.0 |
| $^{90}$Y-folate | 5 | 21.5 | 22.0 |

## 6. Therapy Experiments

**Purpose:** Therapy experiments were performed to investigate the tumor growth delay in each of the treated groups of mice and compare it with untreated controls. Moreover, the body weight was monitored and blood plasma parameters assessed for each mouse after euthanasia.

**Methods:** The detailed methods of monitoring mice regarding the tumor growth and body weights are described in the main article. When an endpoint criterion was reached, blood was sampled from the retrobulbar vein immediately before euthanasia and plasma parameters indicative of kidney or liver damage were measured.

**Results:** The tumor growth curves of each group, presented as the average tumor volume of all mice per group as well as for each single mouse, are shown in Figure S3.

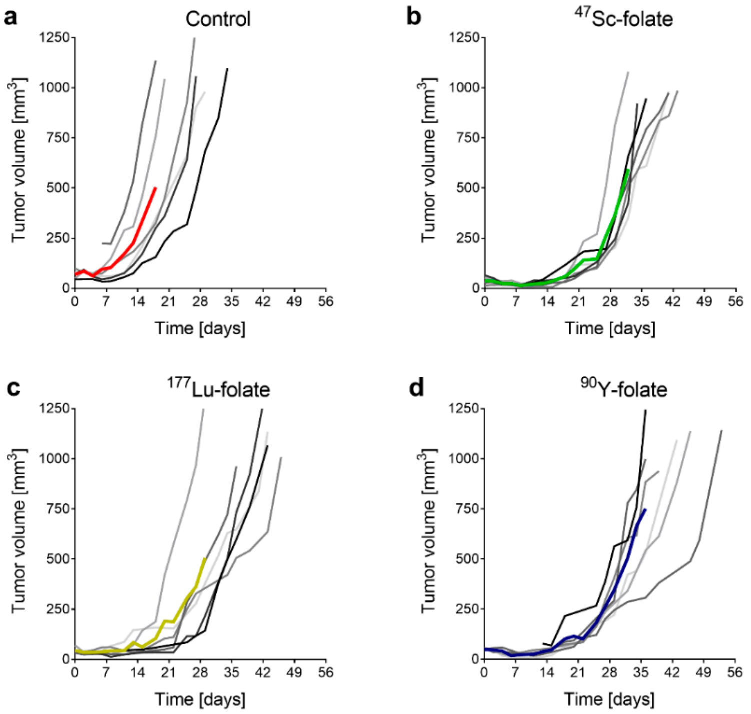


**Figure S3.** Absolute umor volumes of IGROV-1 tumor-bearing mice which received (**a**) saline or ~21 Gy mean absorbed tumor dose of (**b**) $^{47}$Sc-folate, (**c**) $^{177}$Lu-folate or (**d**) $^{90}$Y-folate.

The relative body weights (RBW) of each group, presented as the average RBW of all mice per group as well as for each single mouse, are shown in Figure S4.

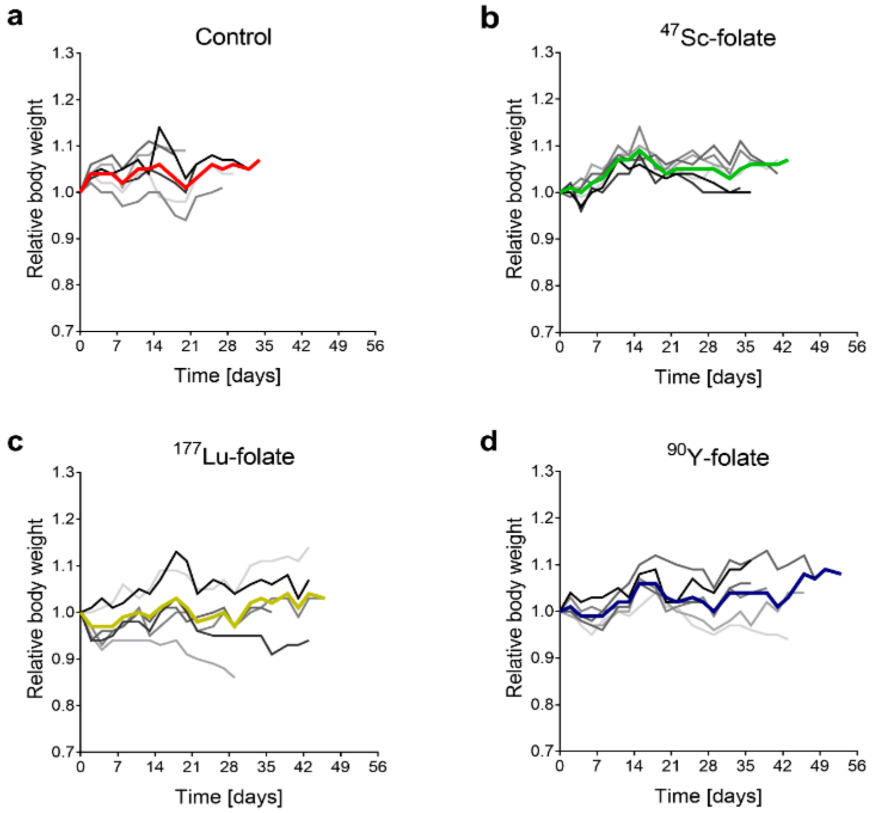


**Figure S4.** Relative body weight of IGROV-1 tumor-bearing mice which received (**a**) saline or ~21 Gy mean absorbed tumor dose of (**b**) $^{47}$Sc-folate, (**c**) $^{177}$Lu-folate or (**d**) $^{90}$Y-folate.

Creatinine (CRE) levels were below the detection limit in all investigated animals (Table S4). Blood urea nitrogen (BUN) levels were comparable among all mice (5.2–7.2 mmol/L). The alkaline phosphatase (ALP) levels were in the same range (40 ± 6 to 57 ± 10 U/L) for all mice and the same held true for total bilirubin (TBIL) levels (3.3 ± 0.8 to 5.5 ± 1.1 µmol/L) (Table S4).

**Table S4.** Plasma parameters measured in the blood plasma of mice at the endpoint.

| Radioconjugate | Activity [MBq] | CRE (µmol/L) | BUN (mmol/L) | ALP (U/L) | TBIL (µmol/L) |
|---|---|---|---|---|---|
| - | - | <18.0 | 5.2 ± 1.1 | 57 ± 10 | 4.2 ± 1.2 |
| $^{47}$Sc-folate | 12.5 | <18.0 | 6.1 ± 1.1 | 46 ± 12 | 5.5 ± 1.1 |
| $^{177}$Lu-folate | 10 | <18.0 | 7.2 ± 3.3 | 43 ± 17 | 3.3 ± 0.8 |
| $^{90}$Y-folate | 5 | <18.0 | 6.6 ± 1.7 | 40 ± 6 | 3.3 ± 0.8 |

## 7. Toxicity Assessment

**Purpose:** Non-tumor-bearing mice were euthanized 2 weeks after radiofolate injection at two different activity levels, followed by assessment of the histopathology. The aim was to determine potential short-term histological changes in the kidneys, bone marrow and spleen.

**Methods:** Non-tumor-bearing mice were injected intravenously with saline or with different activities of $^{47}$Sc-folate, $^{177}$Lu-folate and $^{90}$Y-folate, respectively (Table S5). Body weights were measured twice a week. All mice were euthanized two weeks after the injection of the respective radiofolate. Detailed methods of the pathohistological assessment are reported in the main article.

**Table S5.** Injected activities of the radionuclide in question and molar amounts of the folate conjugate.

| | Activity / Quantity | | Activity / Quantity | |
|---|---|---|---|---|
| | (MBq) | (nmol) | (MBq) | (nmol) |
| Saline | - | - | - | - |
| $^{47}$Sc-folate | 12.5 | 1 | 25 | 2 * |
| $^{177}$Lu-folate | 10 | 1 | 20 | 1 |
| $^{90}$Y-folate | 5 | 1 | 10 | 1 |

* Two nmol were injected because 12.5 MBq/nmol was the highest achievable molar activity for the preparation of $^{47}$Sc-folate.

**Results:** The results of the scoring as a measure of potential side effects are listed in Table S6 and the results are reported in the main article. Representative H/E stained kidney sections of each group are shown in Figure S5.

**Table S6.** Off-target toxicity scores in non-tumor-bearing mice which received equal doses of $^{47}$Sc-folate, $^{177}$Lu-folate or $^{90}$Y-folate.

| Radiofolate | Activity [MBq] | Kidney | Bone Marrow | | Spleen |
|---|---|---|---|---|---|
| | | Radiation Nephropathy | Hypo-cellularity | Lymphoid depletion | Extramedullary hematopoesis |
| - (Saline) | 0 | 0 (9/9) | 0 (9/9) | 0 (9/9) | 1 (1/9)<br>2 (6/9)<br>3 (2/9) |
| Average | | 0.0 | 0.0 | 0.0 | 2.1 |
| | | Lower dose level | | | |
| $^{47}$Sc-folate | 12.5 | 0 (6/6) | 0 (6/6) | 0 (6/6) | 1 (1/6)<br>2 (4/6)<br>3 (1/6) |
| Average | | 0.0 | 0.0 | 0 | 2 |
| $^{177}$Lu-folate | 10 | 0 (6/6) | 0 (6/6) | 0 (6/6) | 3 (5/6)<br>4 (1/6) |
| Average | | 0.0 | 0.0 | 0.0 | 3.2 |
| $^{90}$Y-folate | 5 | 0 (8/8) | 0 (8/8) | 0 (8/8) | 1 (1/8)<br>2 (4/8)<br>3 (3/8) |
| Average | | 0.0 | 0.0 | 0.0 | 2.3 |
| | | Higher dose level | | | |
| $^{47}$Sc-folate | 25 | 0 (3/5)<br>1 (1/5)<br>2 (1/5) | 0 (5/5) | 0 (5/5) | 2 (1/5)<br>3 (3/5)<br>4 (1/5) |
| Average | | 0.6 | 0.0 | 0.0 | 3.0 |
| $^{177}$Lu-folate | 20 | 0 (6/6) | 0 (6/6) | 0 (6/6) | 2 (3/6)<br>3 (3/6) |
| Average | | 0.0 | 0.0 | 0.0 | 2.5 |
| $^{90}$Y-folate | 10 | 0 (6/6) | 0 (4/6)<br>1 (2/6) | 0 (6/6) | 1 (5/6)<br>2 (1/6) |
| Average | | 0.0 | 0.3 | 0.0 | 1.2 |

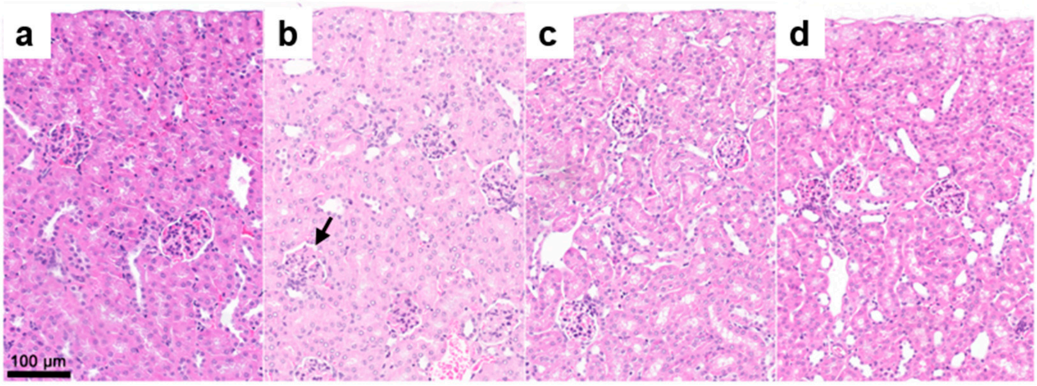


**Figure S5.** Histological findings in the kidney of mice euthanized two weeks after injection of the radiofolates. (**a**) Tissue of a control mouse that received saline; (**b**) Tissue of a mouse treated with 25 MBq $^{47}$Sc-folate (the black arrow shows a focal area of mesangial damage); (**c**) Tissue of a mouse treated with 20 MBq $^{177}$Lu-folate, (**d**) Tissue of a mouse that received 10 MBq $^{90}$Y-folate.

## 8. References

1. Müller, C.; Bunka, M.; Haller, S.; Köster, U.; Groehn, V.; Bernhardt, P.; van der Meulen, N.; Türler, A.; Schibli, R. Promising prospects for $^{44}$Sc-/$^{47}$Sc-based theragnostics: Application of $^{47}$Sc for radionuclide tumor therapy in mice. *J Nucl Med* **2014**, *55*, 1658–1664.
2. Domnanich, K.A.; Müller, C.; Benešová, M.; Dressler, R.; Haller, S.; Köster, U.; Ponsard, B.; Schibli, R.; Türler, A.; van der Meulen, N.P. $^{47}$Sc as useful $\beta^{-}$-emitter for the radiotheragnostic paradigm: A comparative study of feasible production routes. *EJNMMI Radiopharm Chem* **2017**, *2*, 5.
3. Ladino, C.A.; Chari, R.V.; Bourret, L.A.; Kedersha, N.L.; Goldmacher, V.S. Folate-maytansinoids: Target-selective drugs of low molecular weight. *Int J Cancer* **1997**, *73*, 859–864.
4. Siwowska, K.; Haller, S.; Bortoli, F.; Benešová, M.; Groehn, V.; Bernhardt, P.; Schibli, R.; Müller, C. Preclinical comparison of albumin-binding radiofolates: Impact of linker entities on the in vitro and in vivo properties. *Mol Pharm* **2017**, *14*, 523–532.
5. Haller, S.; Reber, J.; Brandt, S.; Bernhardt, P.; Groehn, V.; Schibli, R.; Müller, C. Folate receptor-targeted radionuclide therapy: Preclinical investigation of anti-tumor effects and potential radionephropathy. *Nucl Med Biol* **2015**, *42*, 770–779.
